# Analysis of reactive oxygen and nitrogen species generated in three liquid media by low temperature helium plasma jet

**DOI:** 10.1038/s41598-017-04650-4

**Published:** 2017-07-04

**Authors:** Julie Chauvin, Florian Judée, Mohammed Yousfi, Patricia Vicendo, Nofel Merbahi

**Affiliations:** 1 Université de Toulouse; UPS, INP; LAPLACE, 118 route de Narbonne, F-31062 Toulouse, France; 2 CNRS; LAPLACE, F-31062 Toulouse, France; 3 Laboratoire des IMRCP, UMR CNRS 5623, Université de Toulouse, 31062 Toulouse, France

## Abstract

In order to identify aqueous species formed in Plasma activated media (PAM), quantitative investigations of reactive oxygen and nitrogen species (ROS, RNS) were performed and compared to Milli-Q water and culture media without and with Fetal Calf Serum. Electron paramagnetic resonance, fluorometric and colorimetric analysis were used to identify and quantify free radicals generated by helium plasma jet in these liquids. Results clearly show the formation of ROS such as hydroxyl radical, superoxide anion radical and singlet oxygen in order of the micromolar range of concentrations. Nitric oxide, hydrogen peroxide and nitrite-nitrate anions (in range of several hundred micromolars) are the major species observed in PAM. The composition of the medium has a major impact on the pH of the solution during plasma treatment, on the stability of the different RONS that are produced and on their reactivity with biomolecules. To emphasize the interactions of plasma with a complex medium, amino acid degradation by means of mass spectrometry was also investigated using methionine, tyrosine, tryptophan and arginine. All of these components such as long lifetime RONS and oxidized biological compounds may contribute to the cytotoxic effect of PAM. This study provides mechanistic insights into the mechanisms involved in cell death after treatment with PAM.

## Introduction

Low temperature plasmas generated at atmospheric pressure have been studied in the past few years for their applications in the field of medicine and biomedicine. Short and long lived reactive oxygen (ROS) and nitrogen species (RNS) can be generated by non-thermal plasmas in either gaseous or aqueous forms when primary plasma species (ions, electrons, radicals, and dissociated molecules) interact with a liquid phase^1–3^. It is noteworthy that plasmas can induce either cell proliferation for low doses or cell death by apoptosis for high doses of exposure^4, 5^. Low temperature plasmas have therefore been studied intensively for wound healing^6^, sterilization^7^, blood coagulation^4^, dental treatment^8, 9^ and also for the inactivation of various cancer cells from breast^10^, head and neck^11^, ovarian^12^, lung^13^, prostate^14^ or colorectal tissues^15–17^. It has been shown that plasma species can inactivate cancer cells either directly (interactions of gaseous species with cells) or indirectly when using a previously-prepared plasma-activated liquid media (PAM). In addition, such plasma treatments can selectively inactivate cancer cells without really affecting normal cells^16, 18–20^. Interestingly, PAM have numerous advantages: i) they allow selective treatment of internal organ cancer tissues which are difficult to reach by the gaseous species and requiring endoscopes or catheters; ii) they present minimal toxicity for normal tissues; and iii) they remain stable several days after their preparation if they are stored at the right temperature^13, 16, 21^. In PAMs, reactive oxygen and nitrogen species (RONS) have been shown to induce cancer cell apoptosis^11, 12^, although the cell death pathways at a molecular level have not yet been clearly elucidated, some studies have suggested mitochondrial dysfunction^13, 22^. Identification and quantification of the aqueous RONS generated in PAMs could therefore shed light on the mechanisms of action of PAM with regard to tumor eradication and wound healing. We have already described the genotoxic and cytotoxic effects of PAMs on colon adenocarcinoma multicellular tumor spheroids and its selective action on cancer cells elsewhere^16, 20^.

Furthermore, most of plasma devices described in the literature which are used to generate PAMs are based on dielectric barrier discharge setups (DBD) using RF, AC or pulsed power supplies with different carrier gases such as helium^10, 15, 16^, helium with oxygen^11, 14^ or argon^12, 13^. The nature and quantity of the plasma species generated depend on the type of plasma device used and the carrier gas composition. Hence, there is a real demand for the identification and quantification of the aqueous RONS (superoxide anion radical, hydroxyl radical, singlet oxygen, nitric oxide, hydrogen peroxide, nitrite/nitrate, etc.) generated when the gaseous plasma species impact the liquid media. Several techniques are currently used to identify and quantify the gaseous plasma products, including optical emission spectroscopy (OES) or laser induced fluorescence^23–25^. However, aqueous plasma by-products with a short lifetime are difficult to quantify using OES^15, 26^, and other methods including chemical dosimetry or fluorescent probes^27, 28^ electron paramagnetic resonance (EPR) spectroscopy^29–33^ appear more appropriate. The later technique requires specific spin traps to allow detection of some aqueous plasma by-products^31^.

The overall goal of this study is the analysis of several plasma-induced free radicals in three liquid media, i.e. Milli-Q water and a cell culture medium, DMEM, with and without fetal calf serum (FCS), which are exposed to a low temperature plasma jet generated by a DBD setup using helium carrier gas at atmospheric pressure^26^. These different liquid media are activated by the plasma jet using different exposure times. The RONS investigated are hydrogen peroxide (H_2_O_2_), hydroxyl radical (^•^OH), singlet oxygen (^1^O_2_), superoxide radical (O_2_^•−^), nitric oxide (NO^•^) and nitrite/nitrate anions (NO_2_/NO_3_). H_2_O_2_ was measured using a fluorometric kit and NO_2_/NO_3_ with a colorimetric kit, while the three other free radicals were quantified using EPR spectroscopy and spin traps due to their short life spans.

## Results and Discussion

### Hydroxyl Radical (^•^OH) produced in liquid media by He plasma jet

**Figure 1:**
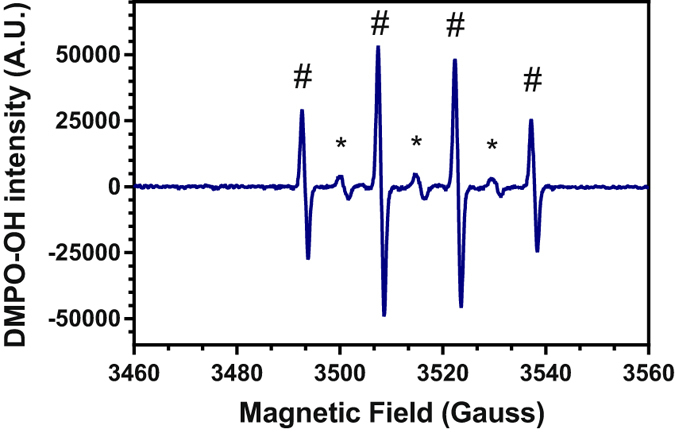
EPR spectrum of DMEM exposed upon 150 s to cold plasma in the presence of DMPO (^#^DMPO-OH, ^*^DMPO-CH_3_).

In order to characterize the formation of reactive oxygen species in different media such as Milli-Q water, a cell culture medium, DMEM, without or with fetal calf serum (FCS) upon exposure to cold plasma, spin trapping experiments were carried out using 5,5-dimethyl-1-pyrroline N-oxide (DMPO) as spin trap. Figure 1 shows the electron paramagnetic resonance (EPR) spectrum of DMEM containing DMPO exposed to the plasma jet for 150 s. Whatever the medium, the EPR spectrum recorded corresponds to the superposition of two signals. The first and the most intense signal yielded the following hyperfine coupling constants a_N_ = a_H_ = 15 G, g = 2.0056 and peak intensity ratio of 1:2:2:1 (Fig. 1, #) which is attributed to DMPO-OH adduct^3, 30, 31, 33–37^. The second signal (Fig. 1, *) is a triplet having a_N_ = a_H_ = 15 G, g = 2.0056 and peak intensity ratio of 1:1:1. This signal is also observed in untreated media (Milli-Q water or DMEM +/− FCS) (data not shown). Under similar experimental conditions, this second signal was also been observed by Tresp *et al*.^34^ and was assigned to DMPO-CH_3_ adduct. DMPO-CH_3_ spin adduct can only originate from the spin trap itself since no carbon-based molecules are present in Milli-Q water. This assumption seems to be confirmed by the absence of correlation between the signal intensity and the exposure time of the media to the plasma jet.

^•^OH radical can be formed by the reaction of an oxygen atom with an H_2_O molecule at the liquid surface^38, 39^ or from the solvation of gaseous ^•^OH produced in the gas phase of plasma giving rise to the production of aqueous ^•^OH radicals. In a previous work, the presence of oxygen atom and OH radical species was validated by observing their specific radiation using emission spectroscopy^15^ This indicates that both pathways may be involved in our experimental conditions. This assumption is supported by the data published by Gorbanev *et al*.^40^ showing that with dry feed He plasma, OH radical may be produced both in the gas phase and in the sample.

**Figure 2:**
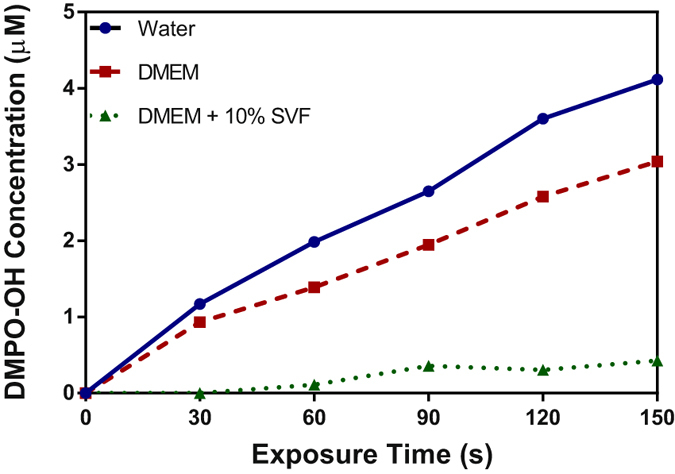
Variation of the concentration of DMPO-OH in water, DMEM + /− 10% FCS as a function of helium plasma jet exposure time. Half height of the second peak of the quartet EPR spectrum was chosen to represent DMPO-OH intensity.

The concentration of OH radical was assessed by exposing the different media with DMPO spin trap to cold plasma. As shown in Fig. 2, the concentration of DMPO-OH adduct increases linearly with the plasma exposure time (R²_water_ = 0.99, R²_DMEM_ = 0.98, R²_DMEM + FCS_ = 0.92) indicating an increase of OH radical production. Kanazawa *et al*.^27^ have already shown similar linear relationship between the amount of OH radical in water and the time of exposure to He plasma jet. For this purpose, they used chemical dosimetry based on the reaction of terephthalic acid with OH radical to generate a fluorescent molecule^27^.

The concentration of DMPO-OH is evaluated around 4.12 µM, 3 µM and 0.4 µM after 150 s exposure to He plasma in water and DMEM +/− FCS, respectively. The amount of DMPO-OH detected in water is higher than in both biological media. Indeed, OH radicals generated in water are surrounded only by water molecules and other types of radicals. In cell culture media, OH radicals can oxidize organic components such as amino acids, vitamins and proteins^41^. These oxidation reactions will reduce the quantities of OH radical trapped by DMPO. Chemical reactions between amino acids and OH radicals were predicted by reactive classical MD simulations^42^ and observed by high-resolution mass spectrometry^43^.

We have estimated the half-life of DMPO-OH in the three media considered Milli-Q water and DMEM +/− FCS. To estimate the half-life of DMPO-OH, the signal decay of DMPO-OH was studied from 120 s to 2000 s after plasma exposure. The data were fitted with a polynomial profile of the 3rd order. The results summarized in Table 1 show that the half-life of DMPO-OH adduct depends on the composition of the medium. In Milli-Q water, the adduct half-life was evaluated at 820 s which is very close to the 870 s found in the literature^31^. In DMEM +/−  FCS the half-life was about 1043 s and 442 s, respectively. The stability of the adduct DMPO-OH is affected by the change in pH and/or the presence of nitric acid^32, 44^. The results suggest that DMEM makes it possible to maintain the pH constant at around 7.4 after exposure to plasma leading to an increase in the stability of the DMPO-OH adduct. In the presence of FCS, its stability is around 2 or 2.3 times lower than in water and DMEM respectively. This might be explained by the fact that the adduct can bind to proteins such as albumin which have documented anti-oxidant properties and/or by the presence of higher quantities of nitric acid than in water and DMEM as we will show in the section nitrite/nitrate anions of manuscript. Nitrite may react with OH radical to form peroxynitrite.

**Table 1:** Half-life of DMPO-OH spin adduct after 150 s of plasma treatment in the three solvents.

Solvent	Half-life DMPO-OH spin adduct (s)
Milli-Q water	820
DMEM	1043
DMEM+ 10% FCS	442

Half height of the second peak of the quartet spectrum was chosen to represent DMPO-OH intensity.

### Formation of Superoxide Anion Radical (O_2_^•−^) by the plasma jet in media

DMPO used as a spin trap in EPR experiments can react with many ROS under different rate coefficients and trapping times. The EPR signal obtained after plasma exposure (Fig. 1) of the different media containing DMPO is clearly defined as the signal of the DMPO-OH spin adduct. However the mechanism is more complex than it seems. DMPO shows a significant preference for the hydroxyl radical (k_OH_ > 10^9^ M^−1^s^−1^ ^31, 34^) but can also scavenge superoxide anion to form the spin adduct DMPO-OOH which has a much lower reaction rate (k_O2−_ < 10^2^ M^−1^s^−1^ ^31, 34^). The difficulty lies in the fact that adduct DMPO-OOH tends to decompose rapidly in DMPO-OH, making it difficult to detect.

**Figure 3:**
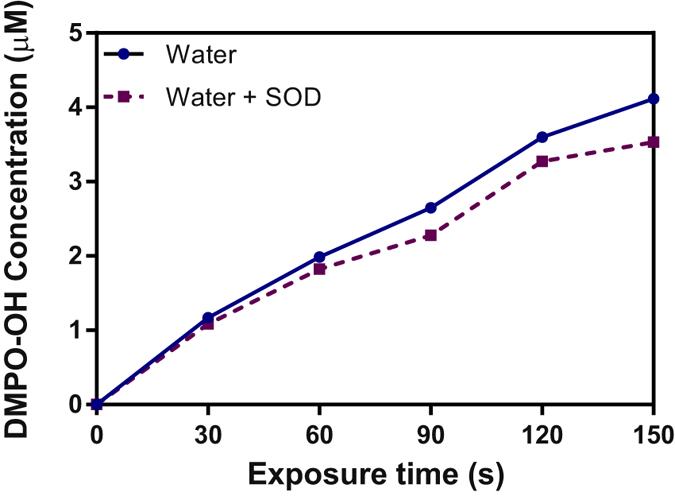
Variation of DMPO-OH concentration in Milli-Q water with or without 150 U of SOD after He plasma treatment at various exposure time. Half height of the second peak of the quartet spectrum was chosen to represent DMPO-OH intensity.

To highlight the presence of superoxide anion in the media after plasma treatment, superoxide dismutase (SOD) was added before plasma exposure. Found in almost all aerobic organisms this enzyme is a metalloprotein that catalyzes the dismutation of superoxide anion radical into molecular oxygen and hydrogen peroxide. The addition of SOD (150 units of SOD per well) to the media leads to the dismutation of the superoxide anion and thus to inhibition of DMPO-OOH spin adduct formation^31^ when exposed to plasma. In water, the addition of SOD leads to a decrease of about 10.2 ± 3.34% in the concentration of DMPO-OH adduct (Fig. 3). This indicates that a small part of the DMPO-OH signal comes from the decomposition of DMPO-OOH into DMPO-OH. It is interesting to note that the amount superoxide anion produced increases linearly with the exposure time. This evolution was also observed by Arjunan and Chyne^45^ using Tempo-9AC (fluorescent probe) and DBD plasma generated in humid ambient air. Superoxide anion is detected in water but in a much lower concentration than OH radical.

### Quantification of the Hydrogen Peroxide (H_2_O_2_) concentration produced in PAM by He plasma jet

H_2_O_2_ is one of the main ROS produced by cold plasmas^13, 16, 20, 33, 40, 45^. As proposed by Gorbanev *et al*., H_2_O_2_ is generated inside the plasma and is delivered into the medium^40^. In our previous work^16, 20^, we showed that hydrogen peroxide produced in PAM remains stable when PAM is stored at 4 °C for at least 7 days. H_2_O_2_ may be considered to be a central genotoxic agent produced during the exposure of media to a He plasma jet. This activity may be attributed to its ability to diffuse through cell membrane and to generate OH radical through a Fenton reaction catalyzed by iron containing proteins. Hydroxyl radical is known for its high reactivity with cellular components, its potent ability to induce DNA damage such as guanine oxidations, DNA cleavage and to cause cell death.

The formation of H_2_O_2_ by He plasma jet was characterized and quantified using a fluorometric Hydrogen Peroxidase Assay kit (procedure described in *Material and Methods*).

**Figure 4:**
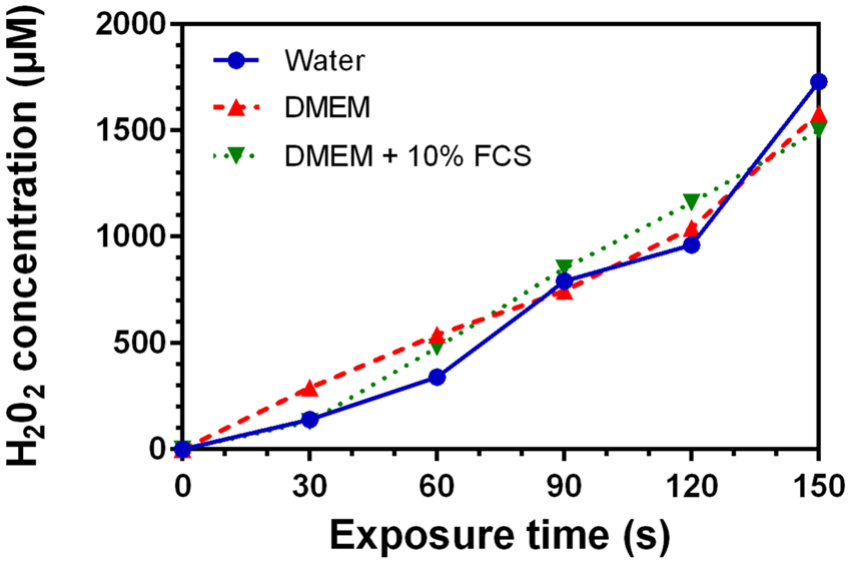
Variation of the concentration of hydrogen peroxide in Milli-Q water, DMEM and DMEM + 10%FCS as a function of He plasma jet time exposition.

As shown in Fig. 4, the H_2_O_2_ concentration in Milli-Q water and cell culture media increases linearly with the exposure time to the plasma jet (R²_water_ = 0.93, R²_DMEM_ = 0.97, R²_DMEM + FCS_ = 0.99). The linear increase in hydrogen peroxide concentration versus exposure time was also reported by Adachi *et al*. using an Argon plasma jet and a DMEM cell culture medium^13^. In the three different media, the hydrogen peroxide concentration can reach 1.60 mM ± 0.12 mM for 150 s of plasma exposure time. This is also very close to the concentration determined in our previous studies^16, 20^. The H_2_O_2_ quantities found are similar to those obtained by Tresp *et al*.using argon plasma to treat saline solution, buffered saline solution and cell culture medium^33^. In contrast to OH radical and superoxide anion radical, the quantities of hydrogen peroxide produced do not depend on the type of the medium treated. This result suggests that H_2_O_2_ will not be consumed *via* a Fenton reaction in DMEM +/− FCS.

### Singlet Molecular Oxygen (^1^O_2_) induced in the liquid media exposed to the plasma jet

It has been shown that singlet oxygen may be generated in water or cell culture media after the treatment of these liquid media by He plasma jet and Air DBD plasma^3, 31, 45^. Wu *et al*. suggested that singlet oxygen was produced in plasma and diffused into the solvent^31^.

**Figure 5:**
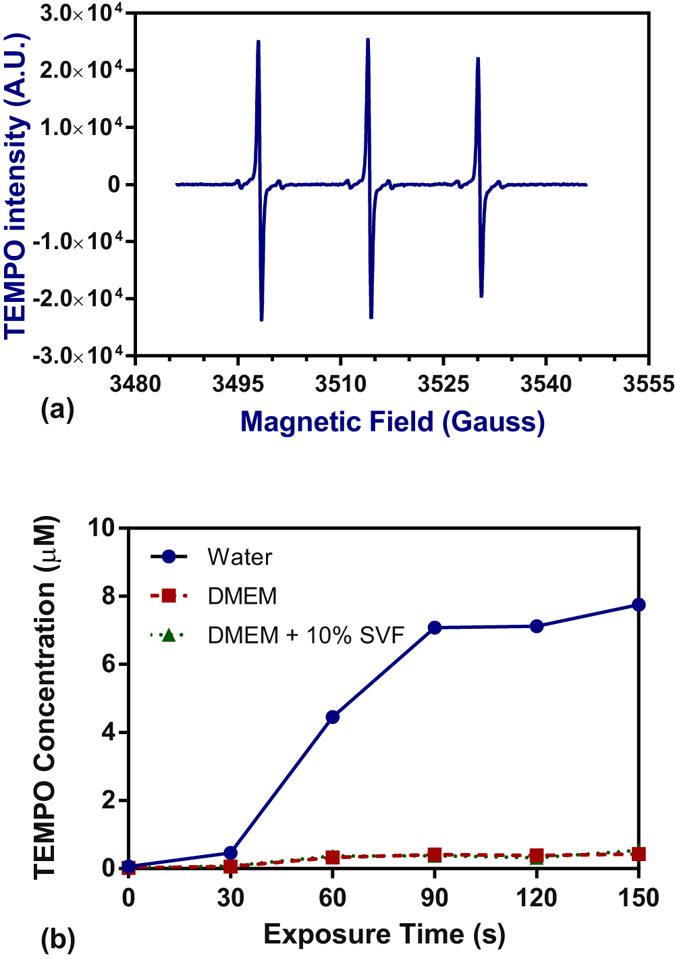
(**a**) TEMPO EPR spectrum obtained after 150 s of plasma treatment in Milli-Q water. (**b**) Evolution of TEMPO signal in three media as a function of plasma jet exposure. Half height of the first peak was chosen to represent TEMPO intensity.

In order to characterize the formation of singlet oxygen in the different media after plasma exposure, EPR experiments were performed using 2,2,6,6-tetramethylpiperidine (TEMP) as the spin trap. As shown in Fig. 5a, the EPR signal of Milli-Q water exposed for 150 s to plasma in the presence of TEMP is characterized by 3 peaks with an intensity ratio of 1:1:1 triplet with hyperfine coupling constants a_N_ = a_H_ = 16 G, g = 2.0059. This signal, detected in the three media, is attributed to the TEMPO spin adducts indicating the formation of singlet oxygen or ozone upon exposure to the He plasma jet. In water, the amount of TEMPO increases linearly with the exposure time (R²_water_ = 0.98, R²_DMEM_ = 0.95, R²_DMEM+FCS_ = 0.88) (Fig. 5b). The addition of NaN_3_ (50 mM), a selective scavenger of singlet oxyimen^46–48^ in water before plasma exposure induced a decrease of around 80% and 51% of the formation of TEMPO after 30 s and 150 s of exposure. This data clearly shows that the formation of TEMPO results from the oxidation of TEMP by singlet oxygen. Moreover, in our plasma jet configuration, with pure He, ozone cannot be detected along the plasma jet by using the UV absorption method at 253.7 nm. This indicates that ozone concentration is very low or negligible. This observation is also in good agreement with the investigations of Kawasaki *et al*.^49^. Both data show that involvement of ozone to the oxidation of TEMPO is negligible in our experimental conditions. The concentration of TEMPO produced in water was evaluated to be about 13.85 µM after 150 s exposure to the plasma jet. The intensity of the TEMPO spin adduct signal in both DMEM and DMEM + FCS is negligible when compared to the case of Milli-Q water. This may be explained by the fact the singlet oxygen is a very high reactive oxygen species known to oxidize cysteine, methionine and tryptophan free amino acids and proteins^50^. Singlet oxygen is consumed in both DMEM and DMEM with FCS. It is worth noting that singlet oxygen contributes to the formation of oxide radicals in PAM leading to a depletion of cellular nutrients. The formation of these oxidized by-products in DMEM +/− FCS may also contribute to the cytotoxic effects of PAM.

### Hydrogen radical (^•^H) induced in liquid media by plasma treatment

**Figure 6:**
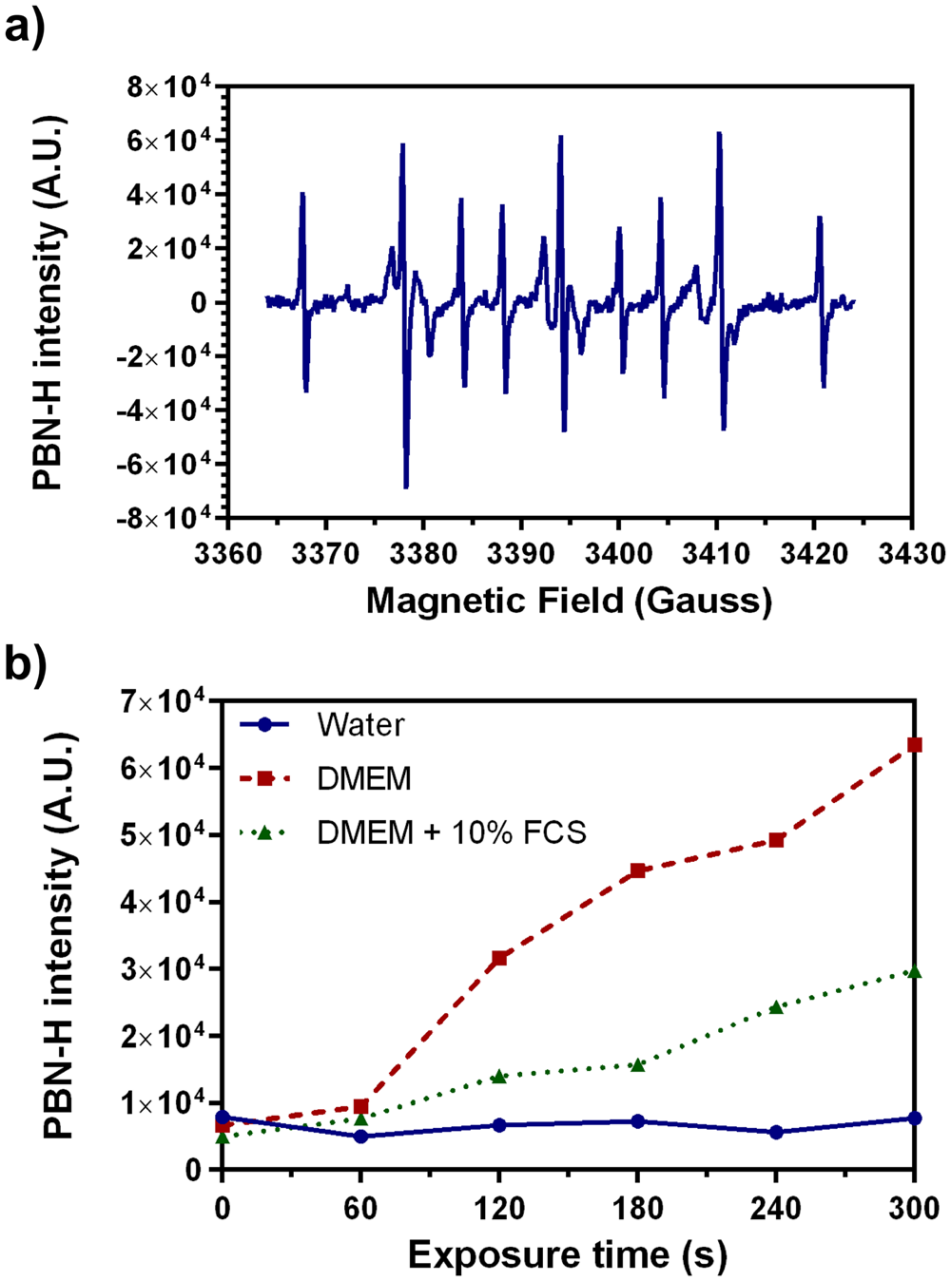
(**a**) PBN-H spectrum obtained after 150 s of plasma treatment in DMEM. (**b**) Effect of plasma exposure on PBN-H signal in three solvents. Half height of the middle peak was chosen to represent PBN-H intensity.

Hydrogen radical was detected by EPR spectroscopy using α-phenyl-N-tert-butylnitrone (PBN) spin trap. The EPR signal obtained during plasma treatment of DMEM is shown in Fig. 6a. This signal is formed by 9 peaks with an intensity ratio of 1:2:1:1:2:1:1:2:1 and with hyperfine coupling constants a_N_ = 16.2 G, a_H_ = 10.3 G, g = 2.0776. It may be attributed to the PBN-H spin adduct. The PBN spin adduct of the ^•^H atom was also observed with cold atmospheric dry-helium^40^ and argon^32^ plasma.

Moreover, a small magnitude signal associated with PBN-H is also observed in Milli-Q water and cell culture media not exposed to the low temperature plasma jet (Fig. 6b at time = 0). Signal intensity was totally independent of exposure time in Milli-Q water whereas it increased linearly in DMEM +/− FCS (R²_DMEM_ = 0.96, R²_DMEM + FCS_ = 0.97). These results suggest that the He plasma jet produces a small quantity of hydrogen radical. This is in total agreement with the spectroscopy studies showing that the He plasma jet generate very small amounts of gaseous H^15^.

In the case of DMEM +/− FCS, the formation of hydrogen radical may be partly attributed to the reaction of ROS such as OH radicals with amino acids^42, 51^. As shown in Fig. 6b, the formation of ^•^H is higher in DMEM than in DMEM + FCS. This difference could be explained by the presence of albumin in DMEM + FCS, a protein known for its antioxidant properties and its ability to trap free radicals^52^. This can lead to a decrease in the amount of ROS able to react with the amino acids in DMEM + FCS.

### Nitric Oxide (^•^NO) formed in liquid media by plasma jet exposure

**Figure 7:**
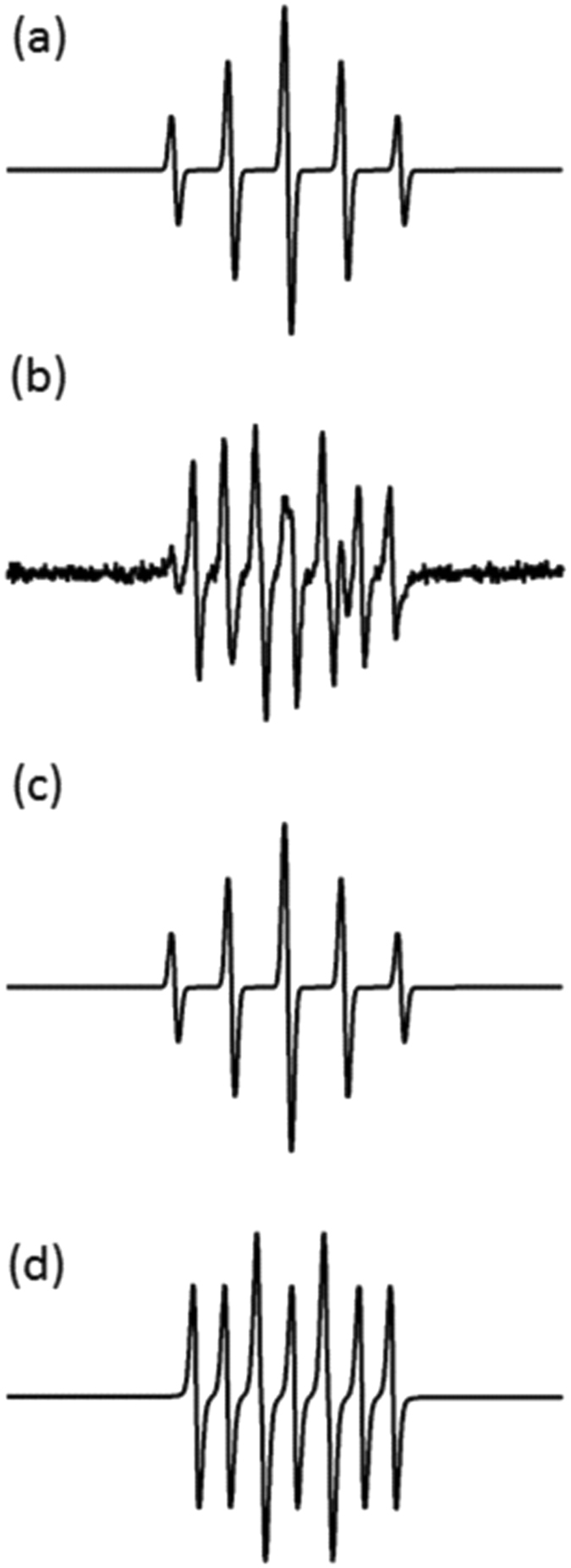
EPR spectra of C-PTIO in Milli-Q water with or without plasma treatment. (**a**) Experimental spectrum of 166 µM C-PTIO in Milli-Q water. (**b**) Experimental spectrum of 16 6 µM C-PTIO in Milli-Q water after 150 s of plasma treatment. Two components are identified in experimental spectrum (**b**) by computer simulation represented in (**c**) and (**d**). (**c**) EPR simulation of C-PTIO identical with spectrum (**a**). (**d**) EPR simulation of C-PTI resulting of the interaction between C-PTIO and nitric oxide.

Nitric oxide was detected by indirect EPR spectroscopy using the Carboxy-PTIO spin trap that can react with nitric oxide to produce Carboxy-PTI and ^•^NO_2._^53, 54^. Carboxy-PTIO and carboxy-PTI are stable molecular radicals that are detectable by EPR spectroscopy and have their own spectral signature. As shown in Fig. 7a the experimental signal of Carboxy-PTIO which is composed by 5 peaks with intensity ratio of 1:2:3:2:1 and hyperfine coupling constants a_N_ = a_H_ = 8.1 G, g = 2.0068 (this signal is obtained without any plasma treatment). Figure 7b shows experimental EPR signal obtained after exposure of 166 µM C-PTIO in Milli-Q water to the He plasma for 150 s. This signal consists of two superimposed radical spectra. The first one was identified as the remaining C-PTIO which does not react with nitric oxide (the simulated spectrum extracted from Fig. 7b is given in Fig. 7c). The second was the EPR signal of C-PTI (the simulated spectrum extracted from Fig. 7b is given in Fig. 7d) and presented 7 peaks with an intensity ratio of 1:1:2:1:2:1:1 and hyperfine coupling constants a_N_ = 9.8 G, a_H_ = 4.4 G, g = 2.0068. The presence of C-PTI in water after plasma exposure confirms the generation of nitric oxide.

**Figure 8:**
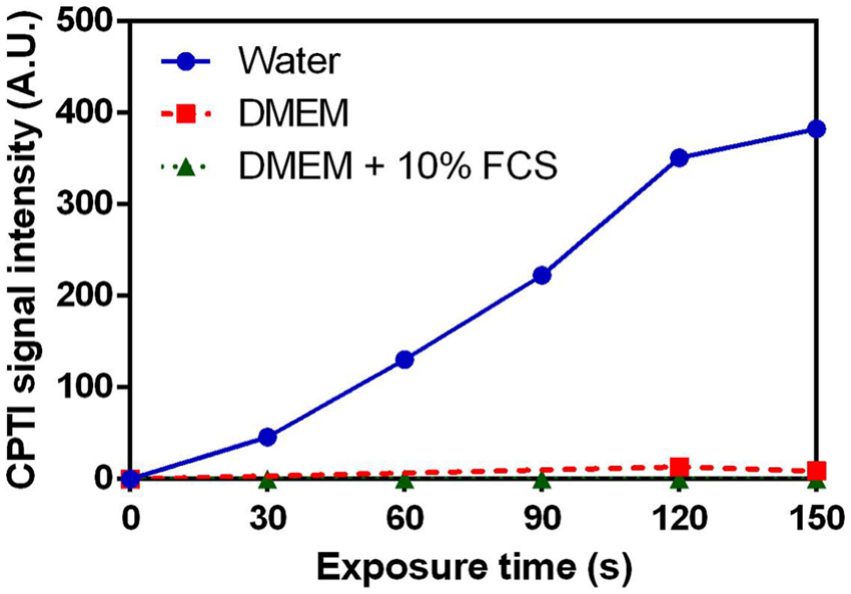
Variation of the concentration of C-PTIin water, DMEM +/− 10% FCS after He plasma treatment at various exposure time.

As shown in Fig. 8, the formation of CPTI in Milli-Q water increases in a linear fashion with (R² = 0.98) exposure to the He plasma jet. The amount of ^•^NO cannot be estimated from the amount of C-PTI formed because C-PTI may be reduced by both hydroxyl and hydrogen radicals to the parent C-PTIO^55^. Nitric oxide in liquids arises after solvation of gaseous nitric oxide produced in the plasma plume observed using emission spectroscopy in our earlier study^15^. Production of nitric oxide in liquids has already been observed in Milli-Q water after its exposure to helium^56^ or argon plasma^35^ and also in buffered saline solution treated with air plasma^57, 58^. In aqueous solution, nitric oxide is a highly reactive species that may react with oxygen to produce nitrite (NO_2_^−^) (Reaction 1)^59^. Formation of NO_2_^−^ in media is known to induce a decrease in the pH of the solution.

1 $${{\rm{4NO}}}^{\cdot }+{{\rm{2O}}}_{{\rm{2}}}+{{\rm{2H}}}_{{\rm{2}}}{\rm{O}}\to {{{\rm{4NO}}}_{{\rm{2}}}}^{-}+{{\rm{4H}}}^{+}$$

**Figure 9:**
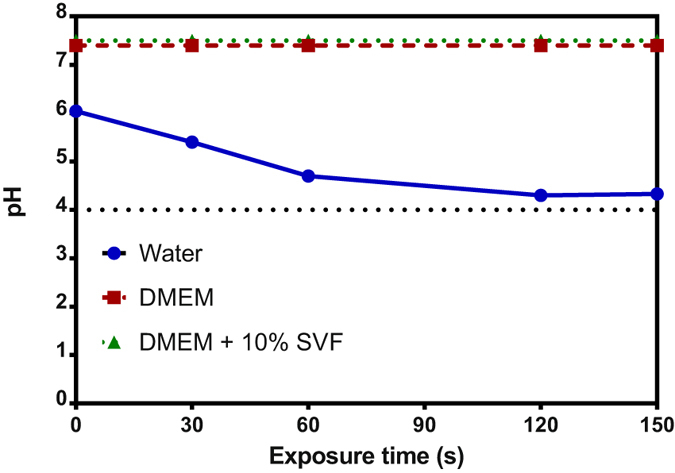
Variation of pH in water after He plasma treatment at various exposure time.

As shown in Fig. 9, we observed a drastic decrease in the pH of water from 6.5 to 4.5 upon plasma exposure. This result suggests the formation of reactive nitrogen compounds such as nitrous acid, nitric acid and peroxynitrous acid^59–62^. Nitrous acid, which is in acidic equilibrium with nitrite, may decompose in acidic medium into nitric oxide and nitrogen dioxide (Reactions 2 and 3)^58^. It is also known that nitrite is not stable under acidic conditions.

2 $${{{\rm{NO}}}_{{\rm{2}}}}^{-}+{{\rm{H}}}^{+}\leftrightarrow {{\rm{HNO}}}_{2}$$

3 $$2{{\rm{HNO}}}_{2}\to {{\rm{NO}}}^{\cdot }+{{{\rm{NO}}}_{2}}^{\cdot }+{{\rm{H}}}_{2}{\rm{O}}$$

In water, nitric oxide may be regenerated *via* reaction 3. In contrast, in cell culture media the amount of NO radical detected is very low. Due to the presence of a buffer, for example phosphate buffer at pH 7.4 in these media, the change of pH will be very slight upon plasma exposure. In phosphate buffer, the pH varies by less than one pH unit^62^ At physiological pH conditions, nitrite will be more stable and subsequently reactions 2 and 3 will not occur or at least be limited in DMEM +/− FCS. This may explain the low concentration of NO in both cell culture media compared to water.

Another possible explanation for the low concentration of NO in biological liquids could be the high reactivity of this radical with bio-macromolecules^63^. NO radicals and related species are able to modify proteins through chemical reactions without involving enzymes. Nitric Oxide groups can bind to a transition metal found in protein or thiol residues of amino acids such as cysteine^64^. Tyrosine is one of the main target of RONS^65^. Nitric oxide can also provide nitrogen sources for the formation of the nitro group (NO_2_). The presence of proteins and amino acids in culture medium can explain the low nitric oxide concentration detected in DMEM +/− FCS.

### Nitrite (NO_2_^−^) and Nitrate (NO_3_^−^) anions generated in liquid media by plasma jet

**Figure 10:**
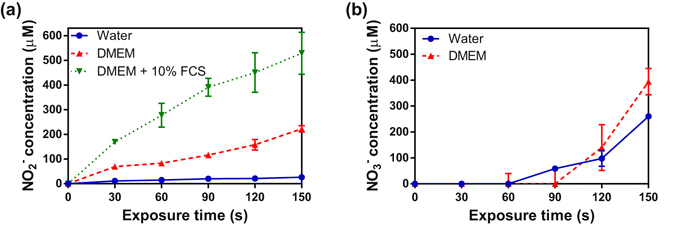
Variation of the concentration of (**a**) Nitrite anion and (**b**) Nitrate anion concentration in water, DMEM + /− 10% FCS after He plasma treatment at various exposure time.

The formation of nitrite and anions during exposure of the different media to a plasma jet was investigated and quantified. The concentration of nitrite in Milli-Q water and culture media (Fig. 10a) increases linearly with exposure time to plasma (R²_water_ = 0.92, R²_DMEM_ = 0.97, R²_DMEM+FCS_ = 0.97). The nitrite concentration in DMEM with fetal calf serum is 2.87 ± 0.47 and 18.77 ± 2.26 times higher than in DMEM and in Milli-Q water respectively.

It is more difficult to quantify the nitrate anion concentration. Figure 10b shows the results in Milli-Q water and serum-free medium only. No anion values can be estimated for plasma jet exposure time of less than 90 s. Above 90 s of treatment, the anion concentration seems to increase linearly with the exposure time. As observed for nitrite, the nitrate anionconcentration is higher in DMEM than in Milli-Q water after 150 s of plasma treatment. Significant uncertainties in the nitrite concentration and the inability to quantify anion in DMEM + 10% FCS could be explained by the interactions between the species produced in this medium by plasma jet and Griess reagent or by the nitrite concentration which is lower than the limit of detection (i.e. 2.5 μM).

Moreover, the negligible values obtained during anion quantification may be due to the non-linearity of the calibration curves (see materials and methods) that increased significantly due to the dilution factor.

Nitrite and anions concentrations in PAM seem to be affected by the composition of the different media. Both nitrogen species are formed in plasma treated media through the dissolution of nitrogen oxides formed in plasma jet. Their formation and their stability in water and DMEM +/−  FCS will depend on different parameters such as the pH of the solution, the presence of amino acids, metallo-proteins and the production of ROS by the He plasma jet. As mentioned above, we have observed in water a drastic decrease in pH, about 2 units, after 150 s exposure to He plasma. In these acidic conditions, nitrous acid (which is one of the major source of nitrite NO_2_^−^) is not stable. It will decompose rapidly into nitrogen dioxide which may subsequently react with hydroxyl radicals produced by the He plasma jet. This chemical reaction leads to the formation of peroxynitrous acid which is not stable in acidic pH and converts into stable nitrate NO_3_^−^. Lukes *et al*.^59^ have shown that, under acidic conditions, nitrite will react with hydrogen peroxide to generate peroxynitrite and subsequently nitrate anion. Girard *et al*.^62^ have clearly identified the formation of peroxynitrite anion in physiological pH under cold atmospheric plasma exposure. This cascade of chemical reactions may explain both the higher level of nitric oxide and the lower quantity of NO_2_^−^ in water than in DMEM +/−  FCS. These reactions will be less efficient in buffered media with a pH of around 7. Hence, the nitrite concentration in DMEM +/−  FCS will be higher than in water. Moreover nitrate/nitrite anions can be the targets of short lifetime ROS such as hydroxyl radical^66^ leading to the formation of peroxynitrite. In complex media such as DMEM +/− FCS, this reaction will compete with the oxidation of biomolecules by ROS. However, the production of peroxynitrite in biological media via this pathway will be minor. Also, the higher concentration of nitrite observed in DMEM + FCS than in DMEM may be explained by the presence of copper proteins such as cytochrome c in FCS that can contribute to the oxidation of nitric oxide into nitrite^67, 68^.

The major source of anion in water and DMEM is the formation of nitrite anion and peroxynitrite. However, anions and nitrite are poorly reactive species with regards to bio-macromolecules. It is mostly ONOO−, NO_2_^•^, N_2_O_3_ that will induce protein and DNA damage such as nitration and nitrosylation of amino acid residuals, nucleic acids and they will be partly responsible for the cytotoxic effect of plasma jet. In DMEM +/− FCS, these RNS will react with the biomolecules causing a decrease in the conversion of peroxynitrite in nitrate anion in these media. Taking into account all the factors influencing the reactivity of RNOS in solutions, the levels of nitrate anion observed should be water> DMEM > DMEM + FCS as was the case here.

It has been shown that nitrate and nitrite anions can be recycled in NO in cells^69^. These inorganic anions produced in PAM are therefore potential sources of NO radicals. Depending on their concentrations, reactive nitrogen species (RNS) are known to have both deleterious and beneficial effects on cell dynamics^70^. Interestingly, the RNS produced by cold atmospheric plasma jets and the quantity produced seem to induce the death of cancer cells in particular offering interesting selectivity between healthy and cancerous cells^20, 70^.

### Plasma treatment of aqueous solutions of amino acids

**Figure 11:**
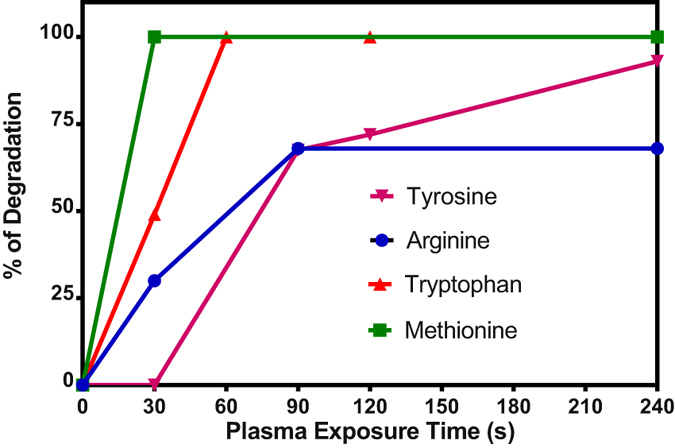
Percentage of degradation of amino acids (0.2 mM) in water after He plasma treatment at various exposure time. The percentage of degradation of each amino acid for an exposure time to He plasma was determined by the following ratio [x−y]/x; where x corresponds to the intensity of the mass peak of the amino acid before treatment and y to the intensity after exposure to He plasma.

Oxidation of aqueous solutions of tyrosine, tryptophan, methionine and arginine after He plasma treatment were studied. The oxidized products were analyzed by HPLC coupled with mass spectrometry (HPLC-QTrap 4500-MS). In addition to the peaks corresponding to the reactants, the mass chromatograms of solutions exposed to plasma showed peaks corresponding to hydroxylation and nitration of tyrosine and tryptophane, sulfoxidation of methionine and hydroxylation of arginine (Figs S1–4 Supplementary data). All of these chemical modifications of amino acids by cold plasma were previously reported by Takai *et al*.^43^. Figure 11 shows that, in descending order, the reactivity of the 4 amino acids is methionine > tryptophan > arginine > tyrosine. Methionine is totally degraded after 30 s exposure to cold plasma. Tyrosine is the only amino acid which is nitrated. All of these data are in accordance with the extensive literature on the reactivity of hydroxyl radical and RNS with amino acids and proteins. These data partially explain the difference in reactivity observed between biological media and water during cold plasma treatment and their different biological activity with regards to cancer cells.

## Conclusion

We have characterized and quantified the formation of radical species such as hydroxyl radical, superoxide anion, singlet oxygen, nitric oxide and long lifetime RONS such as H_2_O_2_ and nitrite/nitrate anions in three culture media (Milli-Q water, DMEM and DMEM with FCS) exposed to a DBD plasma jet using helium at atmospheric pressure as a carrier gas. We have shown that the composition of the medium has a major impact on the pH of the solution during plasma treatment, on the stability of the different RONS that are produced and on their reactivity with biomolecules. Reactions of RONS with DMEM +/− FCS generate oxidized products which may be toxic for cells. Our data indicate that beside the production of long lifetime RONS, oxidized biological compounds form and accumulate in PAM leading to a decrease in essential nutrients for cell growth. All of these components such as long lifetime RONS and oxidized biological compounds may contribute to the cytotoxic effect of PAM previously observed on HCT116 spheroids^16, 20^. Moreover, this suggests that the cytotoxicity of OH radical produced by He cold plasma jet can be mainly due to the production of cytotoxic chemicals in DMEM +/− FCS rather than a direct effect on cellular constituents. Moreover, long lifetime species (H_2_O_2_ and nitrites/nitrates) can penetrate into cells and can be potential precursors of intracellular reactive oxygen species. Indeed, these species can lead in turn to the formation of ^•^OH radical via a Fenton reaction involving H_2_O_2_ and NO^•^ synthesis via recycling of nitrites/nitrates anions by the cells^56^. ^•^OH radical and NO^•^ are highly cytotoxic and genotoxic for cells. Therefore, under the present plasma exposure conditions, the obtained PAM can necessarily generate many potential cytotoxic and genotoxic by-products which have interesting applications, particularly for cancerous cell inactivation.

## Materials and Methods

### Plasma jet device

**Figure 12:**
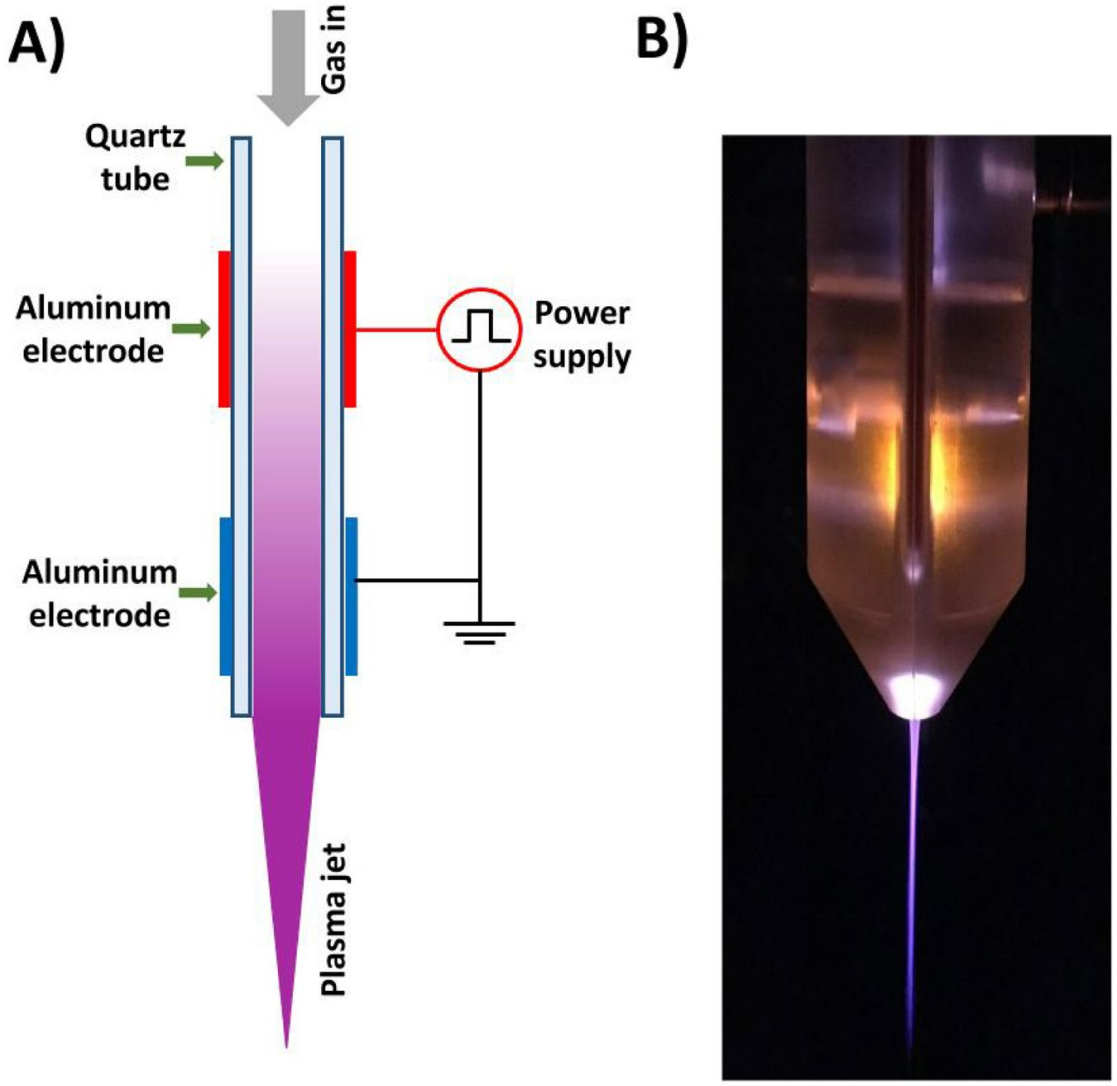
Low temperature plasma jet at atmospheric pressure. (**A**) Schematic diagram of plasma device. (**B**) Picture of plasma jet.

Figure 12 shows a diagram of the plasma jet device based on a dielectric barrier discharge configuration already detailed elsewhere^15, 71^. In short, two aluminum tape electrodes are wrapped around a quartz tube and connected to a High-voltage mono-polar square pulses generator. A power supply with the following characteristics is applied: 10 kV voltage, 9.69 kHz frequency and 1 µs pulse duration. Helium gas flows through the quartz tube at a flow rate of 3 L min^−1^.

### Preparation of Plasma Activated Medium (PAM)

Plasma activated medium (PAM), was produced by exposing 100 µL of water or cell culture medium DMEM with or without fetal to the He plasma jet. These different media were exposed for up to 150 s in 96 well plate, leading to a 20 µl decrease in volume after plasma exposure (data not shown). Plasma exposures were performed under the same experimental conditions (applied voltage, frequency, pulse duration and gas flow) and at the same distance of 2 cm between plasma jet tube output and the upper-surface of the liquid medium.

### EPR spin-trapping spectroscopy

**Figure 13:**
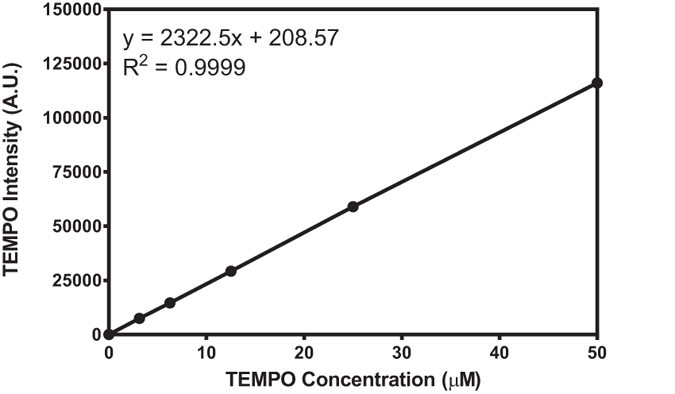
Calibration curve for the analysis of radical adduct using EPR. Double integration of TEMPO EPR spectra versus concentration of TEMPO.

Electron paramagnetic resonance spectroscopy (EPR) is a technique based on the magnetic resonance between an unpaired electron and an external magnetic field. Due to the very short lifetime of radicals, EPR measurement is often used with spin-trap reagents. The reaction of a radical with the spin-trap reagent leads to the formation of a longer-lived spin adduct. EPR spectra were recorded with a Bruker ESP 500E spectrometer at room temperature. The following instrumental settings were employed for the measurements: central field: 3516 G; sweep width: 100 G; microwave frequency: 9.87 GHz; modulation frequency: 100 kHz; microwave power: 5.15 mW; scanning time: 84 s; number of scans: 6. Figure 13 gives EPR calibration performed using an aqueous solution of a stable radical, TEMPO, in concentrations ranging from 0–50 µM^40^.

The spin trapping reagents, 5,5-dimethyl-1-pyrroline N-oxide (DMPO), 2,2,6,6-tetramethylpiperidine (TEMP), α-phenyl-N-tert-butylnitrone (PBN), 2-(4-Carboxyphenyl)−4,5-dihydro-4,4,5,5-tetramethyl-1H-imidazol-1-yloxy-3-oxide potassium salt (C-PTIO), D-Mannitol (OH* scavenger), and superoxide dismutase (superoxide anion scavenger) Sodium azide (NaN_3_) were purchased from Sigma-Aldrich.

Reactive oxygen species such as hydroxyl radical (^•^OH) superoxide anion (0_2_^•−^), singlet oxygen (^1^O_2_), nitric oxide radical (NO^•^) and others including hydrogen radical (H^•^) are presumed to be produced in the different media upon their exposure to plasma. To characterize their formation, the spin trapping reagents DMPO (252 mM), TEMP (2 mM), PBN (21.6 mM) and C-PTIO (833 µM) were added to the different samples immediately before plasma treatment. All samples were transferred in to glass capillary tube (50 µL) immediately after plasma exposure.

To confirm the formation of O_2_^•−^, similar EPR–spin trapping experiments were performed in the presence of SOD (150 U/ml), enzyme catalyzing the conversion of O_2_^•−^ into H_2_O_2_ and O_2_ and directly related to the intensity of the DMPO-OH EPR signal. The DMPO-OH intensity was estimated directly from the half-height of the second peak of the quartet EPR spectra obtained for different media.

All measurements were performed in triplicate for each sample and the changes in the EPR signal were monitored for 2000 seconds after plasma exposure. Easyspin (MATLAB library)^72^ and WinSim2002 software were used for EPR spectra simulations [available online https://www.niehs.nih.gov/research/resources/software/tox-pharm/tools].

### Hydrogen peroxide (H_2_O_2_) assay

**Figure 14:**
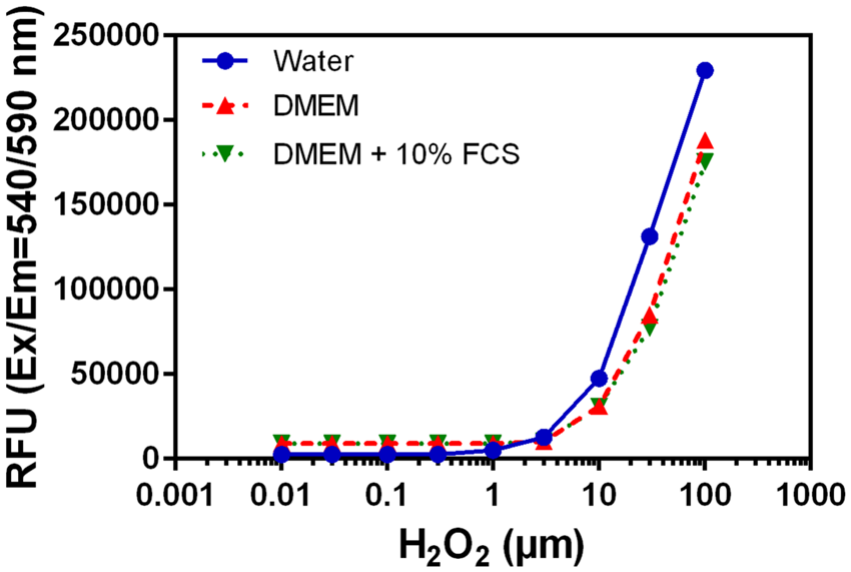
Calibration curves of hydrogen peroxide concentration in three solvents.

H_2_O_2_ concentrations in PAM were quantified using a fluorometric Hydrogen Peroxidase Assay kit (Sigma–Aldrich Co., Ltd). This kit uses horseradish peroxidase and a red fluorescent peroxidase substrate (λex: 540 nm, λem: 590 nm) and allows quantification of H_2_O_2_ in a range of concentrations between 0 and 10 µM. PAM were diluted 1/50 before each measurement in order to achieve an adequate concentration of H_2_O_2_. Calibration curves (see Fig. 14) were plotted for the different liquid media used for the preparation of PAM from an initial 3% hydrogen peroxide solution to avoid any influence of medium absorption on the fluorescence measurements. Samples in black 96-well plates were analyzed using a CLARIOstar fluorescence plate reader (BMG LABTECH) at room temperature.

### Detection of Nitrite/Nitrate anions (NO_2_^−^/NO_3_^−^)

**Figure 15:**
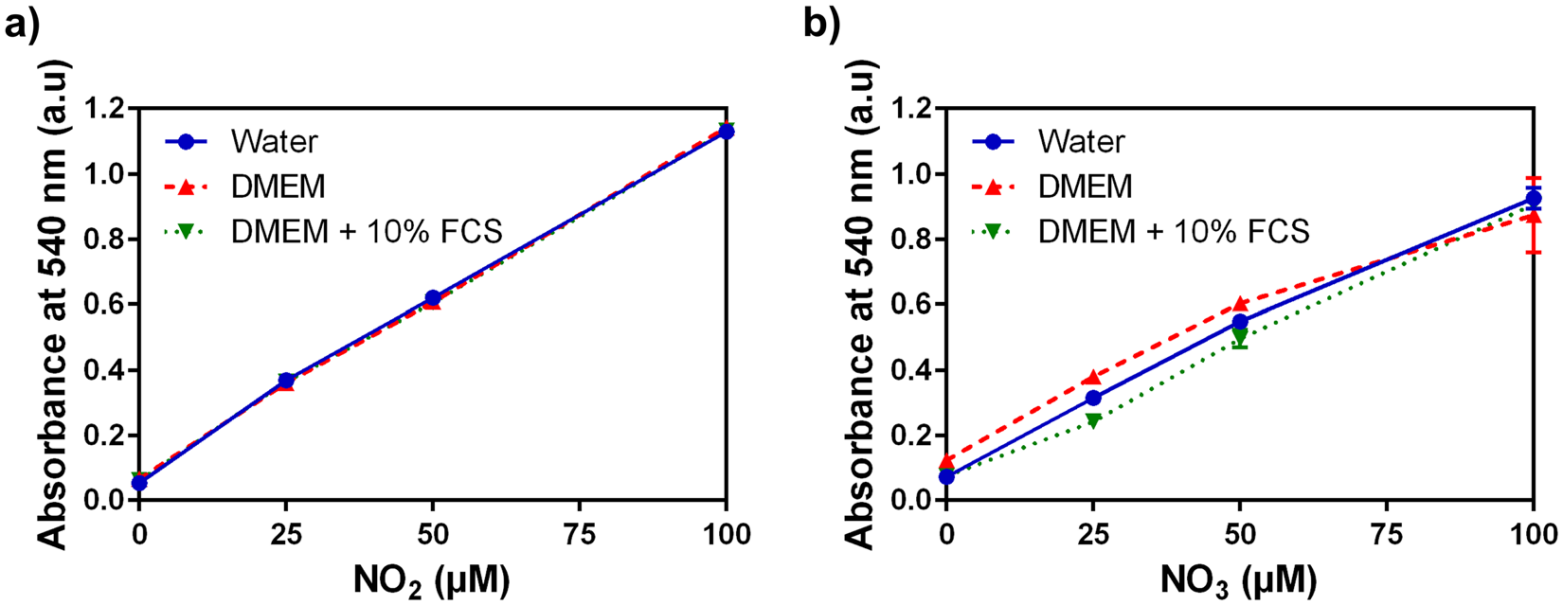
Calibration curves of (**a**) Nitrite anion and (**b**) Nitrate anion concentration in three solvents.

NO_2_/NO_3_ concentrations in PAM were assayed with a colorimetric Nitrite/Nitrate Assay kit (Sigma–Aldrich Co., Ltd) using Griess reagent and nitrite reductase. The kit was initially planned for measurements in a range of concentrations between 0 and 100 µM of NO_2_^−^ and NO_3_^−^. PAM were diluted 1/20 before each measurement in order to have an adequate concentration. Calibration curves (see Fig. 15) were plotted for each medium from initial solutions of NaNO_2_ and NaNO_3_ to take into account the influence of the medium absorption on the measurement. Sample absorbance at 540 nm was analyzed using a CLARIOstar plate reader (BMG LABTECH) at room temperature.

### Mass spectrometry analysis

The LC/MS system was equipped with an HPLC chromatograph (HPLC Agilent 1100 series) and a triple quadrupole mass spectrometer (QTRAP Applied Biosystems). HPLC analyses were performed using a Waters X Bridge C18 (3.5 µm) column (2.1 × 150 mm), and a gradient elution starting with 5% acetonitrile and 95% ammonium acetate at a flow rate of 0.6 mL min^−1^ rising at 15 min a plateau corresponding to 50% acetonitrile and 50% ammonium acetate for 5 minutes. The mass spectrometer was equipped with an electrospray ion (ESI) source (turbo ion spray (TIS) and was operated in positive mode. Nitrogen served as auxiliary, collision gas, and nebulizer gas. The detection was scan mode with a step size of 0.1 atomic mass unit (amu) and a scan range of 50–500 amu. Mass chromatograms, i.e., representations of mass spectrometry data as chromatograms (the x-axis represents time and the y-axis represents signal intensity), were registered using different scan ranges.

The prepared concentration of each amino acids (tryptophan, tyrosine, methionine, arginine) was 0.2 mM in MilliQwater. Each solution was treated by He plasma at various time of exposure (0–240 s).

## Electronic supplementary material

Supplementary Data
