# Supplementary material for: Analysis of reactive oxygen and nitrogen species generated in three liquid media by low temperature helium plasma jet
Source: Sci Rep. 2017 Jul 4;7:4562. doi: 10.1038/s41598-017-04650-4 (PMC5496897; doi:10.1038/s41598-017-04650-4)
Supplement: Supplementary file 1 — Supplementary Data [file 41598_2017_4650_MOESM1_ESM.doc]

**Supplementary Data**

**Analysis of reactive oxygen and nitrogen species generated in three liquid media by low temperature helium plasma jet**

**Julie Chauvin^1,2,3^, Florian Judée^1,2^ , Mohammed Yousfi^1,2^, Patricia Vicendo^3^, Nofel Merbahi^1,2,*^**

^1^ Université de Toulouse ; UPS, INP ; LAPLACE; 118 route de Narbonne, F-31062 Toulouse, France.

^2^ CNRS ; LAPLACE ; F-31062 Toulouse, France.

^3^ Laboratoire des IMRCP, UMR CNRS 5623, Université de Toulouse,  31062 Toulouse, France.

*To whom correspondence should be addressed: [merbahi@laplace.univ-tlse.fr](mailto:merbahi@laplace.univ-tlse.fr)


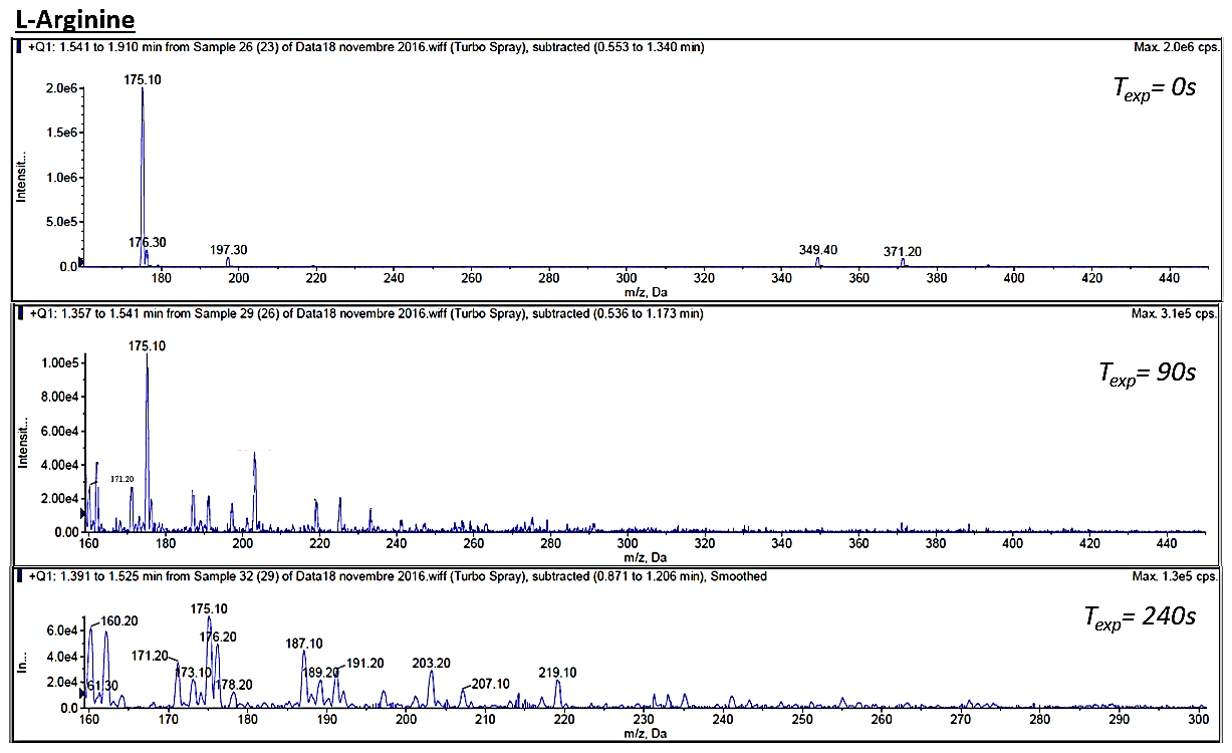


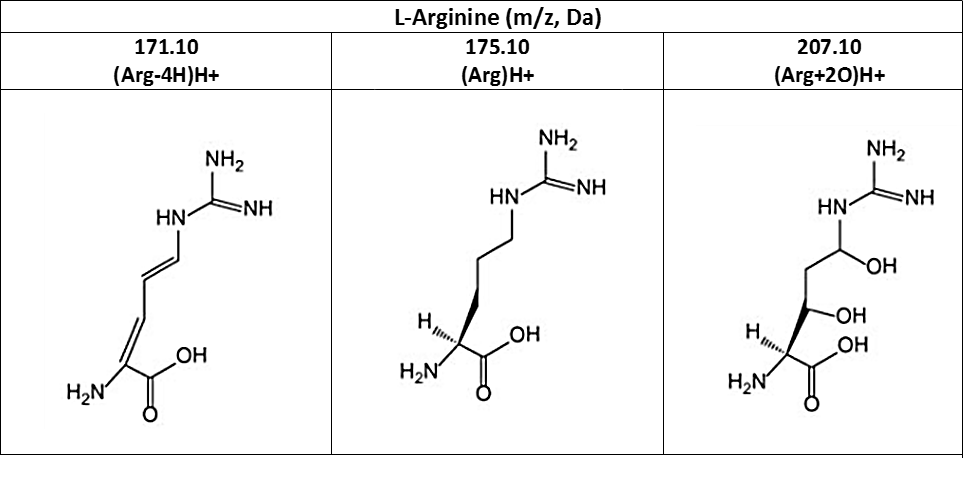


Figure S1: Mass spectra of Arginine treated with He plasma for 0s, 90s,240s. A molecular structure of major products from arginine plasma degradation is proposed.


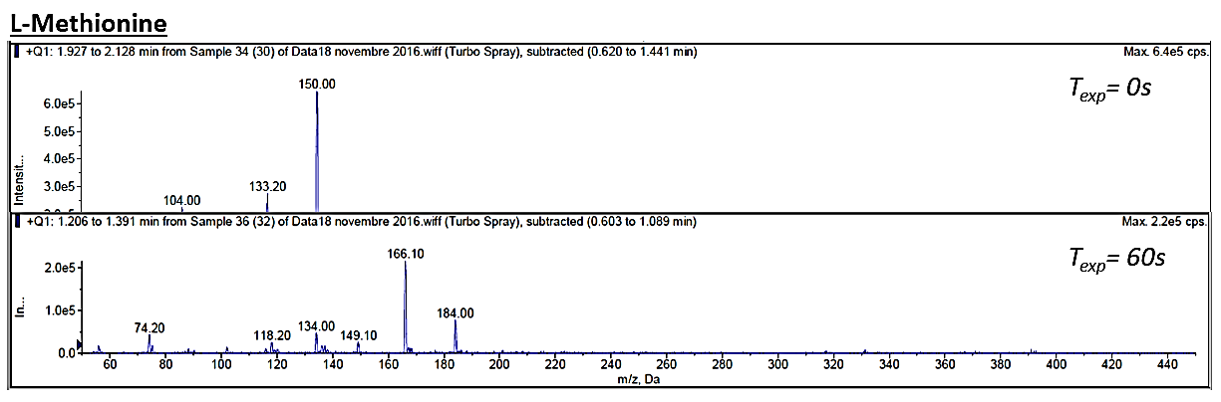


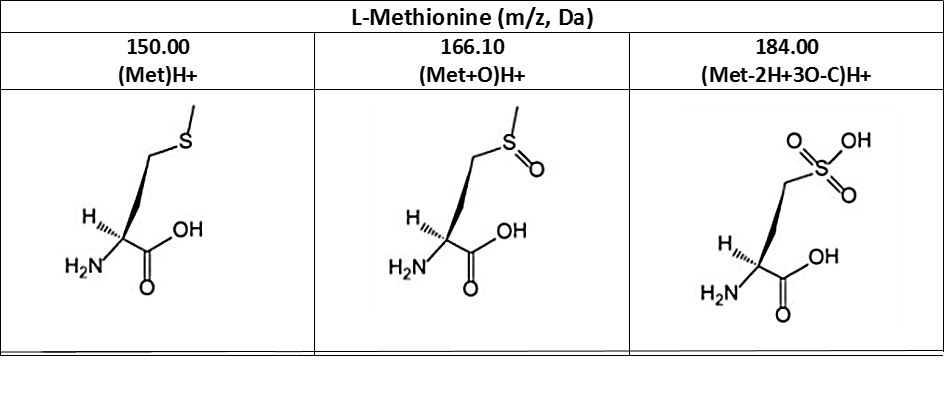


Figure S2: Mass spectra of methionine treated with He plasma for 0s, 60s. A molecular structure of major products from methionine plasma degradation is proposed.


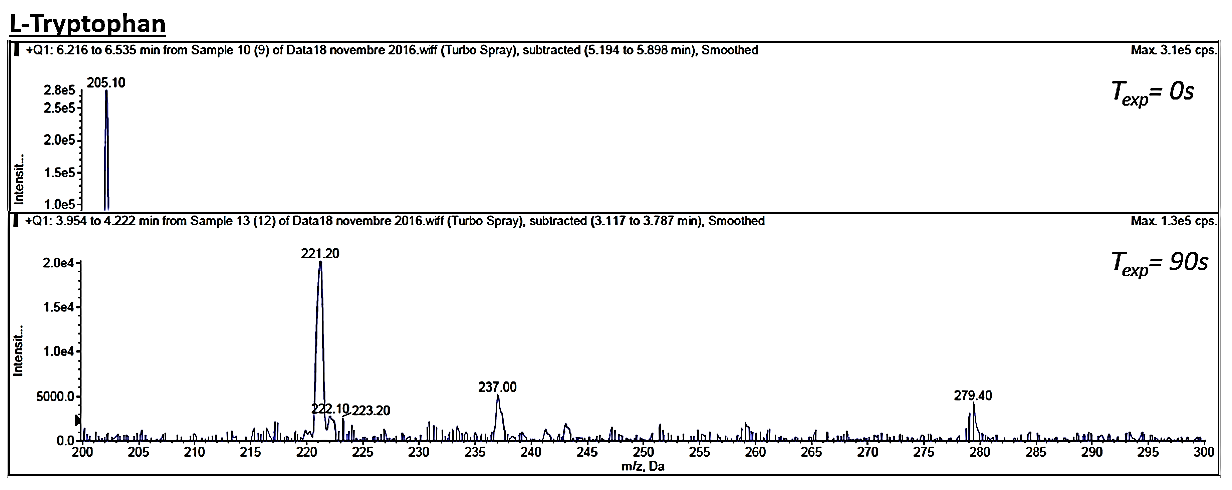


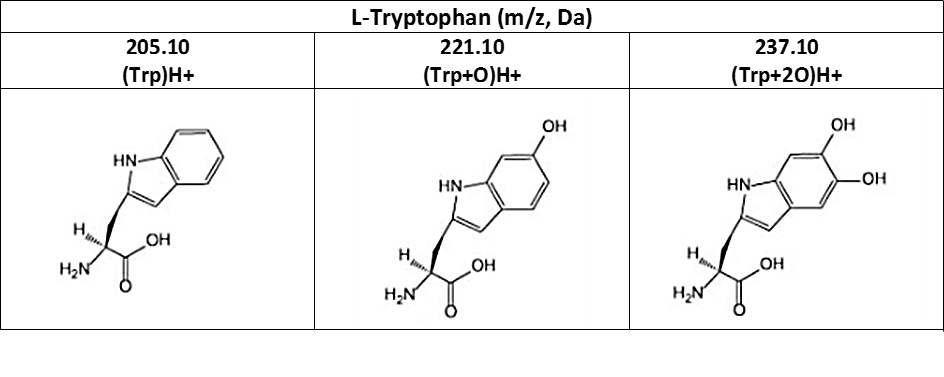


Figure S3: Mass spectra of tryptophan treated with He plasma for 0s, 90s. A molecular structure of major products from tryptophan plasma degradation is proposed.


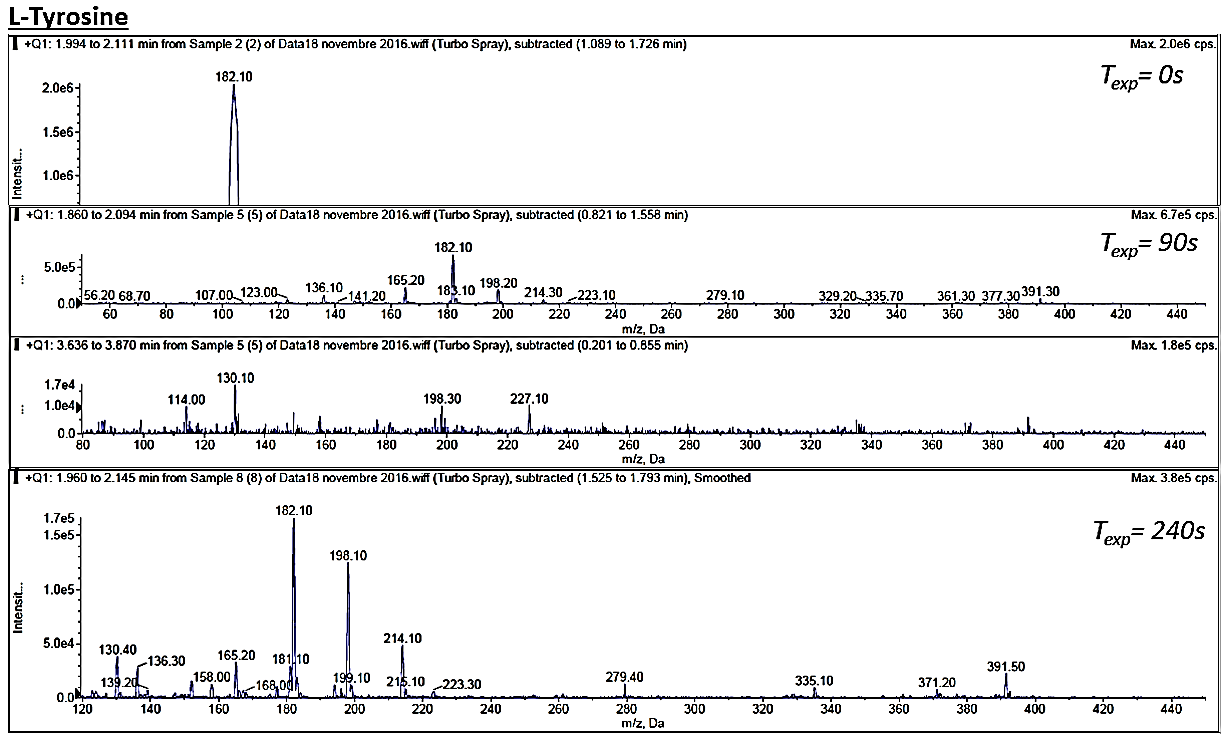


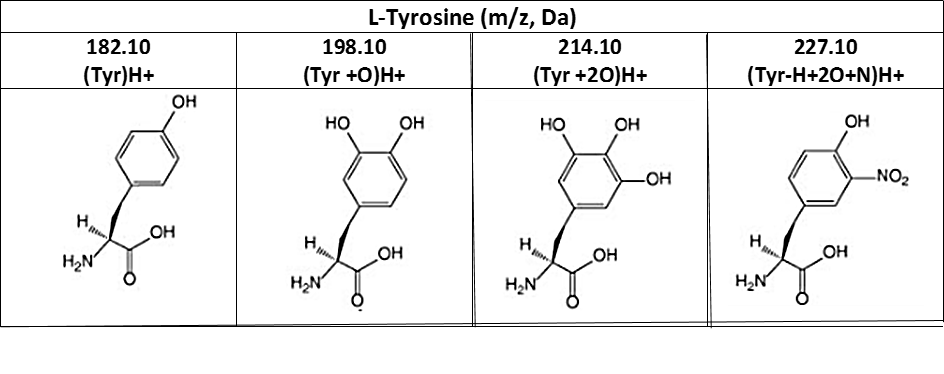


Figure S4: Mass spectra of tyrosine treated with He plasma for 0s, 90s,240s. A molecular structure of major products from tyrosine plasma degradation is proposed.
